# Medical Classes

**Published:** 1845-04

**Authors:** 


### MEDICAL CLASSES.

The following particulars of the number of students at several eastern medical schools during the past winter we derive from the Medical Examiner: viz—

| | |
|---|---|
| University of Pennsylvania, | 446 |
| College of Physicians and Surgeons, New York, | 190 |
| Geneva College, | 183 |
| Harvard University, Boston, | 157 |
| Berkshire Medical Institution, | 145 |

From the published catalogue of the Jefferson Medical College we learn that the number in that institution was 409. Deducting from this, 60 M.D.'s, the number of students, or undergraduates, left is 349. As we have not yet seen the catalogue of the University of Pennsylvania we are not informed how many graduates were in that class. The catalogue of Transylvania University shows 156 names. In this list there are 7 M.D.s, to which another ought to have been added, but, through mistake, no doubt, the title was not appended to the name of S. N. McMinn, a graduate, and he appears among the "practitioners." These 8 subtracted will leave 148 as the number of students, which does not vary from our statement made some months ago. Y.

